# Interstitial Photodynamic Therapy Using 5-ALA for Malignant Glioma Recurrences

**DOI:** 10.3390/cancers13081767

**Published:** 2021-04-07

**Authors:** Stefanie Lietke, Michael Schmutzer, Christoph Schwartz, Jonathan Weller, Sebastian Siller, Maximilian Aumiller, Christian Heckl, Robert Forbrig, Maximilian Niyazi, Rupert Egensperger, Herbert Stepp, Ronald Sroka, Jörg-Christian Tonn, Adrian Rühm, Niklas Thon

**Affiliations:** 1 Department of Neurosurgery, University Hospital, LMU Munich, 81377 Munich, Germany; stefanie.lietke@med.uni-muenchen.de (S.L.); michael.schmutzer@med.uni-muenchen.de (M.S.); c.schwartz@salk.at (C.S.); jonathan.weller@med.uni-muenchen.de (J.W.); sebastian.siller@med.uni-muenchen.de (S.S.); Joerg.Christian.Tonn@med.uni-muenchen.de (J.-C.T.); 2 German Cancer Consortium (DKTK), Partner Site Munich, 81377 Munich, Germany; Maximilian.Niyazi@med.uni-muenchen.de; 3 Department of Neurosurgery, University Hospital Salzburg, Paracelsus Medical University Salzburg, 5020 Salzburg, Austria; 4 Laser-Forschungslabor, LIFE Center, University Hospital, LMU Munich, 81377 Munich, Germany; max.aumiller@med.uni-muenchen.de (M.A.); christian.heckl@med.uni-muenchen.de (C.H.); Herbert.stepp@med.uni-muenchen.de (H.S.); ronald.sroka@med.uni-muenchen.de (R.S.); adrian.ruehm@med.uni-muenchen.de (A.R.); 5 Department of Urology, University Hospital, LMU Munich, 81377 Munich, Germany; 6 Institute for Clinical Neuroradiology, University Hospital, LMU Munich, 81377 Munich, Germany; robert.forbrig@med.uni-muenchen.de; 7 Department of Radiation Oncology, University Hospital, LMU Munich, 81377 Munich, Germany; 8 Center for Neuropathology and Prion Research, University Hospital, LMU Munich, 81377 Munich, Germany; rupert.egensperger@med.uni-muenchen.de

**Keywords:** malignant glioma, glioblastoma, recurrence, photodynamic therapy, 5-ALA, stereotactic surgery, outcome

## Abstract

**Simple Summary:**

Malignant glioma has a poor prognosis, especially in recurrent situations. Interstitial photodynamic therapy (iPDT) uses light delivered by implanted light-diffusing fibers to activate a photosensitizing agent to induce tumor cell death. This study examined iPDT for the treatment of malignant glioma recurrences. Forty-four patients treated at one institution were retrospectively analyzed and patient-, tumor- and treatment-related factors were retrieved from hospital charts. Most of the patients (37) had glioblastomas, the most aggressive type of glioma. Brain swelling or small bleedings caused worsening of symptoms in 18 patients, but only in one case severe symptoms persisted for more than six weeks. After iPDT, tumors recurred after a median of 7.1 months and patients lived for a median of 13.0 months. Two years after iPDT treatment, 25% of the patients were still alive. These promising results should be evaluated further in a prospective study.

**Abstract:**

Interstitial photodynamic therapy (iPDT) using 5-aminolevulinic acid (5-ALA)-induced protoporphyrin IX (PpIX) as a cytotoxic photosensitizer could be a feasible treatment option for malignant gliomas. In a monocentric cohort of consecutive patients treated between 2006 and 2018, a risk profile analysis of salvage iPDT for local malignant glioma recurrences and associated outcome measures are presented here. It was considered indicated in patients with circumscribed biopsy-proven malignant glioma recurrences after standard therapy, if not deemed eligible for safe complete resection. A 3D treatment-planning software was used to determine the number and suitable positions of the cylindrical diffusing fibers placed stereotactically to ensure optimal interstitial irradiation of the target volume. Outcome measurements included the risk profile of the procedure, estimated time-to-treatment-failure (TTF), post-recurrence survival (PRS) and prognostic factors. Forty-seven patients were treated, of which 44 (median age, 49.4 years, range, 33.4–87.0 years, 27 males) could be retrospectively evaluated. Recurrent gliomas included 37 glioblastomas (WHO grade IV) and 7 anaplastic astrocytomas (WHO grade III). Thirty (68.2%) tumors were O-6-methylguanine-DNA methyltransferase (MGMT)-methylated, 29 (65.9%)—isocitrate dehydrogenase (IDH)-wildtype. Twenty-six (59.1%) patients were treated for their first, 9 (20.5%)—for their second, 9 (20.5%)—for the third or further recurrence. The median iPDT target volume was 3.34 cm^3^ (range, 0.50–22.8 cm^3^). Severe neurologic deterioration lasted for more than six weeks in one patient only. The median TTF was 7.1 (95% confidence interval (CI), 4.4–9.8) months and the median PRS was 13.0 (95% CI, 9.2–16.8) months. The 2- and 5-year PRS rates were 25.0% and 4.5%, respectively. The treatment response was heterogeneous and not significantly associated with patient characteristics, treatment-related factors or molecular markers. The promising outcome and acceptable risk profile deserve further prospective evaluation particularly to identify mechanisms and prognostic factors of favorable treatment response.

## 1. Introduction

Despite recent advances in the treatment of malignant glioma, prognosis remains poor with a median overall survival (OS) of less than two years in IDH-wildtype glioblastomas [1,2]. Upon inevitable tumor recurrence, median post-recurrence survival (PRS) is limited to 7–12 months in most recent studies [3,4,5,6,7,8,9,10,11,12]. Salvage treatment options include resurgery, reirradiation protocols, chemotherapy and any combination thereof. Each of these treatment options offers specific advantages and limitations. While salvage surgery can be of advantage in selected cases, only complete resection of the contrast-enhancing tumor mass seems to prolong post-recurrence survival [10,13,14,15]. In the case of eloquent, diffuse and/or multifocal tumor recurrences, reirradiation and/or second-line chemotherapy regimens may be preferable [16,17]. No randomized controlled study, however, has been able to show a relevant survival benefit for any of the treatment regimens tested [18,19,20,21,22,23,24,25]. This also concerns the use of recently introduced tumor-treating fields [6] and some early results from immunotherapy [26]. Accordingly, no standard treatment for progressive/recurrent malignant gliomas exists, and therefore, management has to be optimally tailored to the individual patient [27]. 

Photodynamic therapy (PDT) has been introduced as a local surgical treatment option which is based on cytotoxic effects induced by a photosensitizing agent that is accumulated within malignant glioma tissue and activated by light of an appropriate wavelength [28]. PDT for malignant glioma was developed alongside fluorescence-guided resection with 5-aminolevulinic acid (5-ALA) [29] which provides highly tumor-specific fluorescence in combination with minimal side effects: in an approval study [29] as well as in a more recent assessment [30], safety concerns with respect to drug application or morbidity caused by fluorescence-guided resection did not exceed the ones of standard surgery. PDT can be applied as surface PDT within a resection cavity [31,32,33]. At our institution, PDT is applied as interstitial PDT (iPDT) by cylindrical diffusing fibers that are stereotactically inserted to ensure a spatially precise interstitial irradiation of the target tumor volume. In a former iPDT series of 10 patients with local recurrence of a malignant glioma after multimodal therapy, we demonstrated the feasibility and tolerability of iPDT [28]. In addition, cases of remarkably long tumor control have been observed [34]. Here, results of salvage iPDT of patients (*N* = 44) suffering from local malignant glioma recurrences after standard therapy are presented. Special attention is given to the risk profile of iPDT and associated outcome parameters in this selected patient cohort.

## 2. Patients and Methods

### 2.1. Patient Selection

At our institution, salvage iPDT was considered in adult patients with a Karnofsky performance score (KPS) of at least 70 who presented with a unifocal circumscribed malignant glioma recurrence after standard multimodal therapy. Local tumor recurrence or progressive disease as suspected by contemporary measurements [35,36] must not exceed a maximum extension of 3 cm defined by the contrast-enhancing tumor volume in gadolinium T1-weighted magnetic resonance imaging (MRI). As part of clinical routine, viable tumor tissue was always histologically verified upfront by minimally invasive stereotactic biopsy procedures [37] to exclude treatment-associated effects or pseudoprogression. Use of iPDT was considered indicated in consensus by the neurooncological tumor board when other local therapy options such as (re-)resection or (re-)radiotherapy alone were deemed not to be safely feasible or refused by the patient who qualified for novel experimental treatment options. 

For this single-center retrospective analysis, all 47 consecutive patients who underwent salvage iPDT for malignant glioma recurrences between 2006 and 2018 were included. In three patients, however, no follow-up data were availableAll patients provided written informed consent to treatment. This retrospective investigation was approved by the ethics committee of the Ludwig Maximilian University, Munich, Germany (reference number 19–650).

### 2.2. Magnetic Resonance Imaging Protocol

Magnetic resonance imaging always included three-dimensional gadolinium-enhanced T1- (1 mm slices) and T2-weighted (2 mm slices) scans preoperatively and thereafter. Early postoperative MRI was routinely performed within 48 h after iPDT treatment and additionally included diffusion-weighted imaging (DWI) and apparent diffusion coefficient (ADC) to assess early treatment effects.

### 2.3. Neuropathological Analysis

Neuropathological diagnosis of tumor recurrence was performed according to the respective valid WHO classification from the years 2007 and 2016 [38,39]. After 2009, pyrosequencing was performed routinely to determine isocitrate dehydrogenase (IDH) mutational status and microsatellite analysis for 1 p/19 q codeletion status. In all patients for whom materials were available, IDH mutations were analyzed retrospectively. O-6-methylguanine-DNA methyltransferase (MGMT) promoter methylation was analyzed by methylation-specific PCR and sequencing.

### 2.4. Interstitial PDT Protocol

Interstitial illumination was performed using a diode laser (Ceralas Diode Laser, Biolitec AG, Jena, Germany) and cylindrical diffusing fibers as described before [25]. The target volume was defined after multimodal image fusion of the intraoperative stereotactic CT (contrast-enhanced scans, 0.6 mm axial slices) with the preoperative MRI and, if available, O-(2-[^18^F]fluoroethyl)-L-tyrosine (18F-FET)-PET scan. A spatially precise interstitial irradiation of the individual tumor volumes was planned using the @target 1.19 software (Brainlab, Munich, Germany) [29]. 

The patients were administered 20 mg/kg body weight of 5-ALA three to five hours before the start of iPDT illumination. Depending on tumor geometry, cylindrical diffusing fibers with an active diffuser length of, optionally, 20 mm or 30 mm were selected. The fibers had an outer diameter of 1600 µm (CeramOptec GmbH, Bonn, Germany) (first 26 treatments) and 1560 µm (Light Guide Optics, Rheinbach, Germany) (last 18 treatments), respectively. The fibers were stereotactically inserted with 6 to 12 mm interfiber distances using a modified Riechert stereotactic frame system (Medical High Tech GmbH, Bad-Krozingen-Biengen, Germany). Prior to fiber placement, a mandrin was inserted into the brain along the trajectory to the target to prepare a channel for the fibers and to serve as a reference for an accurate implantation procedure by means of an orthogonal C-arm X-ray evaluation (Siemens Healthineers, Erlangen, Germany). Correct positioning of the light diffusers within the target volume was then ensured using metallic X-ray markers integrated at the distal and proximal ends of the active segment of the cylindrical diffusing fibers. During therapy, patients received mechanical ventilation with 100% oxygen to ensure adequate oxygen supply for the production of free radicals. Interstitial PDT irradiation was performed with a laser light of 635 nm wavelength at the illumination power of 200 mW/cm diffuser length for a treatment time of 60 min. In cases where the treatment plan resulted in small interfiber distances, the illumination power was decreased and the illumination time was prolonged. Illumination was stopped after the scheduled time if no residual fluorescence was observed. Intraoperative spectral online monitoring was performed prior to and after iPDT illumination as described previously [33,40]. In brief, one after the other, each cylindrical diffusing fiber was used as an emitter, while each of the other fibers sequentially served as an optical detection probe connected to a spectrometer (S2000 or USB2000+, Ocean Optics/Ocean Insight, Ostfildern, Germany) measuring the detected light in the wavelength range of 550 nm to 800 nm. After iPDT irradiation, the cylindrical diffusing fibers were removed.

### 2.5. Spectral Online Monitoring Analysis

The acquired spectral online monitoring data were analyzed for detectable transmitted treatment light at 635 ± 3 nm and protoporphyrin IX (PpIX) fluorescence signals at 705 ± 3 nm. To distinguish between signal artifacts and the true signal, a signal threshold of three times the signal-to-noise ratio was assumed. In addition, only fiber pairs less than 19 mm apart were used for spectral analyses. If at least 75% of all transmission or fluorescence signals of a patient showed a true signal, the patient was graded as good, otherwise—as poor.

### 2.6. Perioperative Management and Risk Assessment of iPDT

All iPDT procedures were performed under general anesthesia. In order not to inhibit antitumor immunological effects, no cortisone was administered peri- and postoperatively except in case of uncontrolled edema causing severe neurological deterioration.

Any perioperative sequelae resolving within six weeks were considered transient, all others—as permanent complications of this kind of iPDT treatment. Severity was classified according to the Common Terminology Criteria for Adverse Events Version 5 (CTCAE) [41].

### 2.7. Treatment after iPDT and Follow-Up Evaluation

The decision to perform iPDT as standalone treatment or in combination with additional salvage treatment was made individually by the interdisciplinary neurooncological tumor board considering each patient’s risk profile, previous therapies, as well as molecular tumor characteristics. 

Patients were followed in the outpatient clinic for six weeks after therapy and in three-month intervals thereafter. Radiologic tumor recurrence was defined according to MacDonald or after the response assessment in neuro-oncology (RANO) 2010 criteria [35,36]. Once validated FET-PET data had become available [42,43], FET-PET examination was used, partially in combination with rebiopsy, to rule out pseudoprogression in unclear non-palliative cases.

### 2.8. Statistical Evaluation

The reference point for overall survival (OS) was the date of the initial tumor diagnosis. Time-to-treatment failure (TTF) and post-recurrence survival (PRS) were calculated from the date of iPDT. Patients had been followed until the last patient died. The distribution of continuous (categorical) variables was analyzed by means of the Wilcoxon test (Χ^2^ statistics). Survival was analyzed with the Kaplan–Meier method. A Cox proportional hazards model was used to identify epidemiologic and molecular prognostic factors. Differences between responders and non-responders were analyzed using logistic regression models. The complete statistical analysis was performed with the use of SPSS Statistics 25 (IBM, Armonk, New York, USA). The significance level was set to *p* ≤ 0.05.

## 3. Results

### 3.1. Patient Characteristics

This study included 44 patients (27 (61.4%) males) (Table 1) between 2006 and 2018. The median age at the time of iPDT was 49.4 years (range, 33.4–87.0 years) and the median KPS was 90 (range, 70–100) (Table 1). The median time between initial tumor diagnosis and salvage iPDT was 16.9 months (range, 3.5–192.4 months). Twenty-six (59.1%) patients were treated for their first, 9 (20.5%)—for their second, 9 (20.5%)—for the third or further recurrence. Thirty-six (81.8%) patients had undergone open tumor resection, 43 (97.7%) patients had been treated with percutaneous irradiation and 39 (88.6%)—with chemotherapy before salvage iPDT for circumscrbed tumor recurrences was considered. At the time of salvage iPDT, 37 (84.1%) of the recurrent tumors were classified as WHO grade IV glioblastomas, 7 (15.9%)—as WHO grade III anaplastic astrocytomas. Six (13.6%) patients suffered from a malignant transformation of an initially diagnosed WHO grade II diffuse astrocytoma. Twenty-nine (65.9%) of the tumors were IDH-wildtype tumors, 9 (20.5%) had an IDH mutation. For six patients, residual tumor material was insufficient for retrospective analysis of the IDH status. A methylated MGMT promoter status was recorded for 30 (68.2%) tumors as part of the integrated histopathological diagnosis.

### 3.2. Interstitial PDT Treatment

The median iPDT target volume was 3.34 cm^3^ (range, 0.50–22.8 cm^3^), targeted with a median of 4 (range, 3–8) cylindrical diffusing fibers (Table 2). The minimum irradiation time of 60 min (range, 60–167 min) was received by 29 patients. The median applied light power per cm diffusor length was 200 mW (range, 82–210 mW). The median energy dose applied during iPDT illumination was 8996 J (range, 5760–17,388 J), largely depending on the iPDT target volume and the corresponding adapted number of cylindrical diffusing fibers.

### 3.3. Imging after iPDT

**Figure 1:**
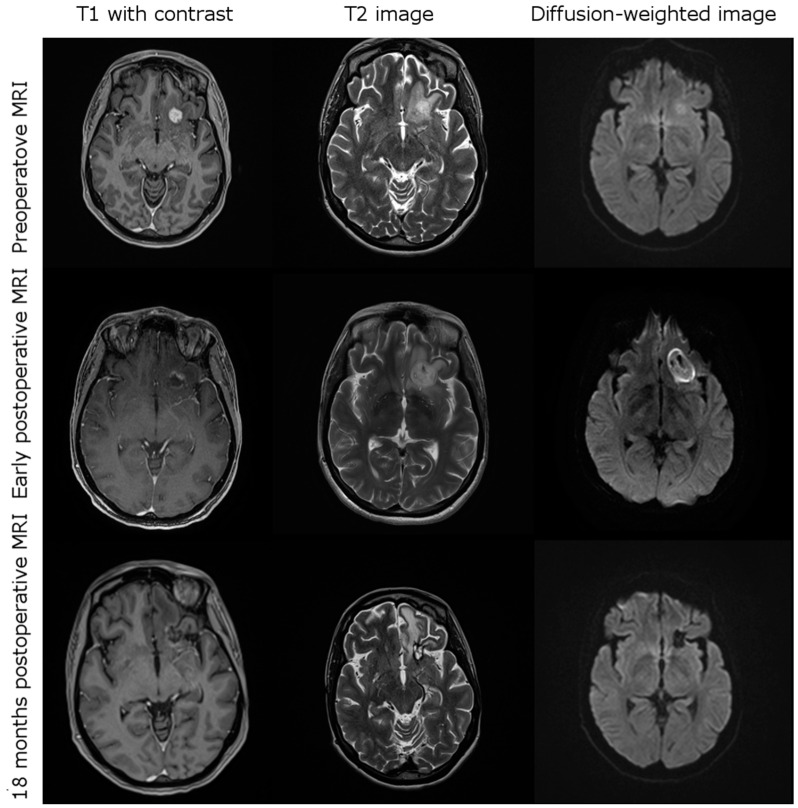
Exemplary case of a 41-year-old female patient suffering from a left frontal glioblastoma recurrence causing mild aphasia. Post-iPDT MRI showing characteristic changes such as disappearance of contrast enhancement, diffusion restriction within the treatment volume and mild perifocal edema. Peri-interventionally, there was transient worsening of aphasia, which then resolved completely in subsequent weeks.

Early postoperative imaging within 48 h after iPDT showed a decrease or effacement of contrast enhancement in all cases. Typically, diffusion restriction occurred in the treated area (Figure 1). A transient increase in edema surrounding the tumor treatment volume was seen in 12 cases, six of which caused transient neurological deterioration (two—paresis, four—aphasia).

### 3.4. Complications after iPDT

Complications after iPDT are listed in Table 3. Overall, 18 patients (40.0%) experienced transient worsening of the usually preexistent neurological deficits, i.e., mainly incomplete aphasia and/or hemiparesis. One patient developed malignant edema and underwent emergency decompression within 24 h after iPDT treatment. In this case, only slight word-finding difficulties (CTCAE °2) persisted. After six weeks, most deficits resolved or would not inhibit activities of daily life (*N* = 9, CTCAE °1). Three patients (6.8%) suffered from residual deficits; in one case, self-care was affected (CTCAE °3). Postoperative complications were not found to correlate with the patients’ TTF (*p* = 0.841) and PRS (*p* = 0.492).

### 3.5. Treatment after iPDT

As part of a predefined combined treatment algorithm, iPDT was immediately followed by chemotherapy in 20 cases (45.5%; temozolomide, *N* = 14, 31.8%; procarbazine/lomustine, *N* = 6, 13.6%) and by re-radiotherapy with/without chemotherapy in 11 (25.0%) cases (Table 2). At the time of tumor progression after iPDT treatment, all patients were found eligible for additional multimodal treatment (31 (70.5%) patients were treated with chemotherapy, 20 (45.5%) received re-radiotherapy and 4 (9.1%) underwent open tumor debulking for space-occupying recurrences before salvage chemotherapy was initiated). No immediately palliative case (usually confined to best supportive care only) was observed. Patients with MGMT-methylated tumors did not receive more or less often radiotherapy (*p* = 0.81) or chemotherapy (*p* = 0.92) in the further course of their disease.

### 3.6. Outcome

**Figure 2:**
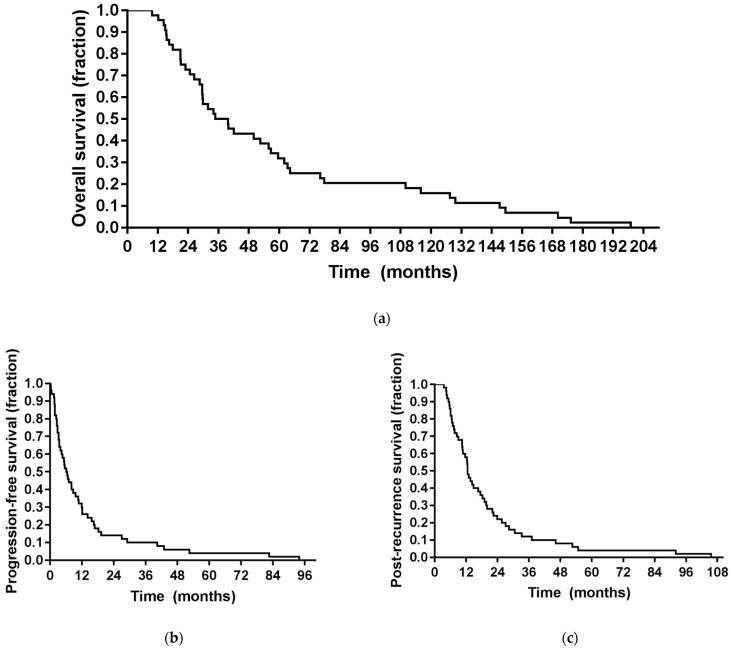
Survival curves. (**a**) Overall survival of the study population after initial tumor diagnosis; (**b**) time-to-treatment failure (TTF) of the study population after iPDT treatment; (**c**) post-recurrence survival (PRS) of the study population after iPDT treatment.

The median follow-up duration was 13.0 months (range, 4.7–105.6 months). In three patients, however, no follow-up data were available. Within their individual follow-up duration, all the remaining patients experienced tumor progression and died, with all recorded deaths being tumor-related. Here, the median OS from the first tumor diagnosis was 39.7 months (range, 9.8–199.0 months). The median time between the first diagnosis and salvage iPDT was 16.9 months (range, 3.5–192.4 months), median TTF after iPDT was 7.1 months (range, 0.6–93.9 months; 95% confidence interval (CI), 4.4–9.8) and median PRS was 13.0 months (range, 4.7–105.6 months; CI, 9.2–16.8) (Figure 2). When the three patients lost to follow-up were also included in the analysis (as part of the intended-to-treat population), median TTF was 6.8 months and mean PRS was 12.5 months (Supplementary Figure S1). As can be derived from Figure 2, six- and twelve-months recurrence-free survival rates after salvage iPDT were 59.1% and 34.1%, respectively. Six- and twelve-months survival rates after iPDT were 88.6% and 63.6%, respectively. Two years after iPDT treatment, eleven (25.0%) patients were alive, seven (15.9%) of them recurrence-free. 

### 3.7. Prognostic Factors

In Table 4, the correlations of prognostic factors with regard to PRS after iPDT are summarized. Univariate analysis showed a significant influence of KPS on PRS (*p* = 0.019). No significant influence of age, MGMT promoter methylation or IDH mutational status, target volume, time to iPDT treatment or any salvage treatment modality on PRS could be found. Time between initial diagnosis and iPDT did not correlate with PRS (Pearson’s coefficient, −0.112, *p* = 0.469) or TTF (Pearson’s coefficient, −0.169, *p* = 0.274). No salvage treatment regimen was associated with an additional survival benefit, neither in the complete study cohort nor in the subgroup of patients with a methylated MGMT promoter sequence. No known prognostic factor was found to correlate with TTF (Supplementary Table S1). FET-PET imaging before treatment was available for 12 patients (median maximum Standardized Uptake Value (SUVmax), 2.95, range, 2.2–8.2) and after treatment for 15 patients (median SUVmax, 2.7, range, 1.5–7.4). Post-therapeutic decrease in SUVmax was associated with longer TTF (*p* = 0.081).

### 3.8. Intraoperative Spectral Online Monitoring

Intraoperative transmission and fluorescence data could be evaluated for 18 patients. A total of 300 pre- and postoperative spectra each were analyzed, with one spectrum recorded for each possible fiber pair. The median number of spectra per patient was 24 (range, 12–84). Overall, insufficient postoperative transmission was detected in 21.7% of the spectra and preoperative fluorescence in 15.8%. Of the 18 patients, 13 (72.2%) showed good postoperative transmission rates between two fibers (of 75% or more), indicating sufficient illumination over the complete duration of treatment. Ten (55.6%) also showed PpIX fluorescence over a threshold of 75%. In three (16.7%) cases, only preoperative fluorescence was graded as good, but not the postoperative transmission. In all patients, no residual PpIX fluorescence could be detected after the iPDT. Good postoperative transmission and preoperative fluorescence were associated with longer TTF and PRS, although the differences were not highly significant (Table 5).

## 4. Discussion

This study’s main findings are: (1) iPDT was technically feasible in all patients; (2) iPDT can be performed with acceptable risk even in highly eloquent tumor localizations; (3) iPDT appears to be associated with favorable treatment effects even in heavily pretreated malignant glioma recurrences; (4) iPDT as part of a salvage treatment concept is associated with considerable long-term survival (PRS > 2 years) in a—so far—poorly defined subpopulation of malignant glioma patients; (5) treatment success was not associated with any of the known conventional and molecular prognostic factors of malignant gliomas. 

Interstitial PDT is a technically demanding form of local treatment that is used for various tumor diseases [44]. Malignant glioma cells are characterized by a selective and effective uptake of systematically administered 5-ALA with subsequent intracellular conversion into the red fluorescent, phototoxic PpIX by exploiting the enzymatic machinery of heme synthesis [45]. As a photosensitizer, PpIX mediates energy transfer from light photons to oxygen molecules to generate reactive oxygen species that lead to oxidation and destruction of membranes, proteins and other vital intracellular structures [46,47]. Some of the cell death mechanisms initiated in this manner effectively stimulate the immune response, adding an intriguing systemic effect to this otherwise local treatment modality [48,49].

These characteristics of 5-ALA in combination with the possibility of a spatially precise interstitial illumination by means of a stereotactic frame-based installation of the respective light diffusers make iPDT an attractive tool for local malignant glioma recurrences after standard therapy. This minimally invasive procedure might be of advantage over the technical alternative of a surface PDT, in which an illumination source is introduced directly into the resection cavity at the end of surgery to illuminate any residual tumor tissue. The disadvantage of this method is that the extent of the residual tumor tissue cannot be determined with certainty and the penetration depth of illumination is limited to a few millimeters. However, since a spatially precise illumination of the target tumor volume seems to be fundamental to achieve full treatment effects, the image-guided stereotactic iPDT may be preferred. Moreover, iPDT is not limited to resectable tumors. Only few centers, however, have gained some preliminary clinical experience with iPDT in malignant brain tumors so far (yet unpublished personal communications). To date, just one prospective study has been published which suggested 5-ALA-based iPDT to be safe and feasible for a selected patient population with glioblastoma recurrences [28]. No permanent procedure-related morbidity was seen in the 10 patients treated and select patients survived surprisingly long, possibly thanks to immunological effects known from PDT treatment [50]. 

This manuscript presents clinical experiences in a large cohort of 44 multimodally pretreated patients who underwent iPDT for small, unifocal and circumscribed malignant glioma recurrences with a maximum extension of 3 cm. This size limitation was based on the maximum number of light fibers per laser and the experimental data, which have shown optimal spacing of light diffusers of about 7–9 mm for accurate tissue illumination without causing critical thermal effects [28].

Based upon intense interdisciplinary and collaborative efforts, dedicated equipment had been made available so that treatment planning, diffusing fiber insertion and light application were technically feasible in all intended cases. By means of the intraoperative spectral online monitoring technique, transmission of treatment light between fibers and PpIX fluorescence light could be monitored. During most treatments, PpIX fluorescence was characterized as good before starting the illumination and vanished during irradiation, which, at least in cases with good postoperative light transmission grading, indicates substantial consumption of PpIX as expected due to photobleaching of this photosensitizer. The technique of spectral online monitoring seems to be a promising tool to monitor 5-ALA administration, PpIX accumulation and fiber placement and potentially to estimate treatment effectiveness. In addition, it was observed that the portion of spectral data indicating good treatment light transmission decreased during treatment. This can be used as an indicator that optical tissue properties change such that the light penetration depth is reduced during iPDT illumination, commonly leading to partial and, in some cases, complete transmission signal loss. The reasons for these changes in the in vivo situation have not yet been investigated and understood in detail. However, this observation is in accordance with a previous study [51], where a reduction of treatment light transmission during iPDT was already reported. 

Transient clinical deterioration was recorded in about 40% of patients and was mainly due to exaggerated edema and/or some hemorrhagic imbibition. The latter observations could not be attributed to direct interference of individual catheter trajectories with intratumoral vascular structures.

Only one patient experienced a grade 3 neurological deterioration persisting for more than six weeks. In this retrospective iPDT study, symptoms usually resolved within six weeks after iPDT even without steroid treatment, which was held back so as not to impede possible immunological effects. The development of perioperative complications was independent of the density of previous tumor treatments, tumor volume and the number of laser fibers implanted. This may be due to the limitation to tumors not exceeding 3 cm in extension suggested by earlier studies [33].

Comparison of these complication rates to those reported after other local treatments is challenging because of differences in patient selection, especially regarding tumor size and potentially eloquent location. In the de novo unselected setting, the current gold standard local treatment, fluorescence-guided resection, has reported adverse events in about 40% of the cases, with grade 3 or worse aphasia in up to 6% and hemiparesis in up to 4% of the treatments [52]. For resection in eloquent location, neurological deterioration in up to 60% of the treatments has been reported [53]. In the recurrent setting, complication rates for open resection have been suggested to even increase sequentially in each recurrence situation [54,55]. As an adjunct or alternative to reresection, re-radiotherapy (with/without bevacizumab) emerged as an increasingly used salvage treatment option after 2011. Notably, toxicity rates of grade III or higher aphasia have been described in between 4% [56] and 32% [57] of the treatments. In summary, in an often multimodally pretreated and symptomatic patient population, any local treatment is associated with an increased risk of at least transient morbidity.

The observed median PRS of 13.0 months is in the upper range of the median PRS originated by other treatment modalities, ranging mostly between seven and nine months [17]. In the DIRECTOR study where first glioblastoma recurrences were treated with different temozolomide dose regimens, PRS ranged between 7.9 months for MGMT-unmethylated and 12.5 months for MGMT-methylated tumors [58]. In that cohort, complete resection of contrast enhancement was associated with a more favorable PRS of 12.9 months, compared to incomplete resection (PRS, 6.5 months) and no resection (PRS, 9.8 months), indicating, like the present study, a benefit of local treatment if possible [10]. 

In the present iPDT cohort, no influence of molecular markers such as MGMT and IDH on response to iPDT with or without adjuvant therapy including temozolomide can be observed. Different numbers and types of pretreatments and a heterogeneous patient population may explain the lack of an observable effect or a still too low total number of cases, as other studies also did not uniformly observe a prognostic effect of MGMT promoter methylation or lack thereof in recurrent situations [58,59]. Due to different treatment groups in different studies, a direct meaningful comparison of outcome data is rather difficult. Interestingly, though, more than 20% of patients in the iPDT series survived more than two years after malignant glioma recurrence irrespective of time to PDT and later salvage treatments and even with MGMT-unmethylated primary glioblastomas. This high percentage of long-term survivors warrants further investigation. As shown, common prognostic factors in malignant glioma patients did not differ between long-term and short-term survivors in this cohort, based on a threshold PRS of two years. Possibly, immunological factors may play a role, as has been suggested in experimental settings [60,61]. One of the key factors stimulating the immune response may be the heat shock protein HSP-70 as it was shown that sublethal 5-ALA PDT upregulated HSP-70 by up to 50-fold in glioma cells [49]. The upregulation of HSP-70 and the fact that 5-ALA PDT on glioma spheroids attracted and matured coincubated dendritic cells was experimentally confirmed [62]. The cell death mechanisms glioblastoma cells in a patient treated with iPDT undergo are expected to be very heterogeneous, as both light distribution and photosensitizer distribution are not homogenous. Therefore, parts of the tumor may undergo direct necrosis, others—apoptotic cell death and some cells in the periphery may survive. However, the plethora of cell death mechanisms initiated may be advantageous for an efficient stimulation of immune response with presentation of damage-associated molecular patterns, production of inflammatory cytokines such as interleukin 6 and attraction and maturation of dendritic cells, altogether leading to recruitment of effector T cells as reviewed in [63]. These authors suggested a combined therapeutic approach of PDT with immune checkpoint blockade. Further research, especially into immunological processes surrounding glioblastoma treatment, is required to gain more detailed and specific knowledge to benefit most from iPDT.

In addition, standard MRI images after iPDT showed loss of contrast and FET enhancement and a change in the diffusion restriction precisely confined to the treatment volume. The interpretation of such findings with regard to mechanisms and clinical relevance will be addressed in future studies. Importantly, data interpretation in this study is not hampered by immunologically induced imaging effects as tumor recurrence was histologically verified in all non-palliative cases.

## 5. Conclusions

Interstitial PDT of gliomas remains a challenging procedure due to limited light penetration depth in brain tissue, a complex planning and implantation procedure and potential risk of clinical deterioration especially after treatment in eloquent areas. Nevertheless, in experienced hands, iPDT may be a promising treatment option in a high-risk patient population combining acceptable, mostly transient morbidity with the possibility of long-term survival. It does not critically interfere with but may rather complement other treatment options in the recurrent disease such as re-radiotherapy and/or salvage chemotherapy regimens. These data strongly support further investigation in a controlled prospective setting.

## Figures and Tables

**Table 1:** Summary of patients’ characteristics.

Characteristics at iPDT	All Patients (*N* = 44)
Age (years)	
Median	49.4
Range	33.4–87.0
Gender, n (%)	
Male	27 (61.4)
Female	17 (38.6)
KPS, n (%)	
100	3 (6.8)
90	29 (65.9)
80	9 (20.5)
70	3 (6.8)
Symptoms, n (%)	
Seizures	27 (61.4)
Aphasia without paresis	12 (27.3)
Paresis without aphasia	4 (9.1)
Aphasia and paresis	1 (2.3) ^1^
Side, n (%)	
Right	20 (45.5)
Left	24 (54.5)
Localization, n (%)	
Deep-seated involvement	31 (70.5)
Eloquent lobar localization	17 (38.6)
Stage of disease, n (%)	
First tumor recurrence	26 (59.1)
Second tumor recurrence	9 (20.5)
Third tumor recurrence	6 (13.6)
Later stages	3 (6.8)
WHO grade, n (%)	
IV	37 (84.1)
III	7 (15.9)
WHO grade at initial diagnosis, n (%)	
IV	30 (68.2)
III	8 (18.2)
II	6 (13.6)
MGMT promoter methylation status, n (%)	
Methylated	30 (68.2)
Unmethylated	14 (31.8)
IDH mutation, n (%)	
Wildtype	29 (65.9)
Mutated	9 (20.5)
Unknown (no material left)	6 (13.6)
1 p/19 q codeletion, n (%)	
1 p/19 q codeletion	1 (2.3)
No 1 p/19 q codeletion	20 (45.5)
Not tested	23 (52.3)

^1^ Percentages may not total 100.0 due to rounding.

**Table 2:** Treatment characteristics.

Treatment Algorithms	All Patients (*N* = 44)
**Treatment before iPDT, n (%)**	
Tumor resection	36 (81.9)
Irradiation	43 (97.7)
Chemotherapy	39 (88.6)
**Interstitial PDT characteristics**	
Target volume (cm^3^)	
Median	3.34
Range	0.50–22.8
Number of cylindrical diffusing fibers (range)	4 (3–8)
Applied light power per diffuser length (mW/cm)	
Median	200
Range	82–210
Treatment dose (J)	
Median	8883
Range	5760–17,388
Treatment time (minutes)	
Median	60 (*N* = 29)
Range	60–167
**Salvage treatment with iPDT, n (%)**	
Chemotherapy	20 (45,4)
Radiotherapy	7 (15.9)
Radiochemotherapy	4 (9.1)
**Treatment after iPDT failure, n (%)**	
Any chemotherapy	31 (70.5)
Any (re-)radiotherapy	20 (45.5)
Any tumor resection/debulking	4 (9.1)

The bold cells are sub-headlines.

**Table 3:** Complications after iPDT.

Complications after iPDT	All Patients (*N* = 44)
**Postoperative CTCAE grade, *N* (%)**	
0	26 (59.1)
1	4 (9.1)
2	10 (22.7)
3	3 (6.8)
4	1 (2.3)
5	0 (0.0)
**CTCAE grade at six weeks, *N* (%)**	
0	32 (72.7)
1	9 (20.5)
2	2 (4.5)
3	1 (2.3)
4	0 (0.0)
5	0 (0.0)
**Type of complications, *N* (%)**	
Aphasia	7 (15.9)
Paresis	6 (13.6)
Paresis and aphasia	4 (9.1)
Hyp-/dysaesthesia only	1 (2.3)
None	26 (59.1)

The bold cells are sub-headlines.

**Table 4:** Prognostic factors for PRS after iPDT in uni- and multivariate models; HR—hazard ratio, CI—confidence interval.

Characteristic	HR	Univariate 95% CI	*p*-Value	HR	Multivariate 95% CI	*p*-Value
**Post-recurrence survival (after iPDT)**						
Age at iPDT	1.006	0.981–1.033	0.635	1.023	0.968–1.080	0.424
KPS at iPDT	0.936	0.886–0.989	0.019	1.961	0.755–5.092	0.167
MGMT methylation	0.751	0.392–1.438	0.388	1.000	0.339–2.946	1.000
IDH mutation	0.878	0.405–1.904	0.742	1.018	0.326–3.178	0.976
Target volume	1.019	0.956–1.086	0.557	1.017	0.943–1.096	0.666
Time to iPDT	1.003	0.996–1.010	0.392	1.003	0.992–1.015	0.553
Further chemotherapy	0.811	0.421–1.561	0.530	1.030	0.443–2.392	0.946
Further radiotherapy	0.999	0.540–1.849	0.999	1.005	0.317–3.189	0.994
Further surgery	0.782	0.276–2.212	0.643	1.303	0.258–6.568	0.748

The bold cells are sub-headlines.

**Table 5:** Results of statistical tests of the association of intraoperative spectral online monitoring with time-to-treatment failure (TTF) and post-recurrence survival (PRS), including 95% confidence intervals (CI).

Spectral Data (*N* = 18)	Median TTF (CI) (Months)	*p*-Value	Median PRS (CI) (Months)	*p*-Value
Transmission < 75%	9.63 (.978; 18.28)		12.97 (12.03; 13.92)	
Transmission > 75%	15.73 (4.71; 24.96))	0.196	19.70 (4.32; 35.08)	0.130
Fluorescence < 75%	9.63 (4.20; 15.06)		16.57 (7.83; 25.31)	
Fluorescence > 75%	12.13 (3.84; 20.42)	0.427	17.47 (4.20; 30.74)	0.326

## Data Availability

No new data were created or analyzed in this study. Data sharing is not applicable to this article.

## Supplementary Materials

The following are available online at https://www.mdpi.com/article/10.3390/cancers13081767/s1, Figure S1: Survival curves. (a) Overall survival of the intention to treat population after initial tumor diagnosis; (b) time-to-treatment failure (TTF) of the intention to treat population after iPDT treatment; (c) post-recurrence survival (PRS) of the intention to treat population after iPDT treatment; Table S1: Prognostic factors for TTF after iPDT in uni- and multivariate models HR—Hazard ratio, CI—confidence interval.
