# Supplementary material for: Interstitial Photodynamic Therapy Using 5-ALA for Malignant Glioma Recurrences
Source: Cancers (Basel). 2021 Apr 7;13(8):1767. doi: 10.3390/cancers13081767 (PMC8067827; doi:10.3390/cancers13081767)
Supplement: Supplementary file 1 [file cancers-13-01767-s001.pdf]

cancers

MDPI

Supplementary Material

Interstitial Photodynamic Therapy using 5-ALA for Malignant Glioma Recurrences

Stefanie Lietke, Michael Schmutzer, Christoph Schwartz, Jonathan Weller, Sebastian Siller, Maximilian Aumiller, Christian Heckl, Robert Forbrig, Maximilian Niyazi, Rupert Egensperger, Herbert Stepp, Ronald Sroka, Jörg C. Tonn, Adrian Rühm and Niklas Thon

Supplementary Table S1. Prognostic factors for TTF after iPDT in uni- and multivariate models HR - Hazard ratio, CI – confidence interval.

	Univariate			Multivariate		
Characteristic	HR	95% CI	P-value	HR	95% CI	P-value
**Time to treatment failure (after iPDT)**						
Age at iPDT	1.001	.977-1.026	.910	1.035	.978-1.094	.236
KPS at iPDT	.973	.940-1.007	.121	.970	.925-1.018	.222
MGMT methylation	.856	.446-1.641	.639	1.221	.386-3.860	.734
IDH mutation	.849	.393-1.835	.677	1.244	.380-4.076	.718
Target volume	1.000	.934-1.071	.998	1.001	.926-1.083	.974
Time to iPDT	1.006	.999-1.013	.118	1.005	.995-1.016	.328
Further Chemotherapy	1.366	.708-2.638	.352	1.023	.439-2.387	.958
Further Radiotherapy	.999	.536-1.860	.997	.662	.205-2.137	.490
Further Surgery	1.131	.400-3.196	.817	1.724	.374-7.950	.485

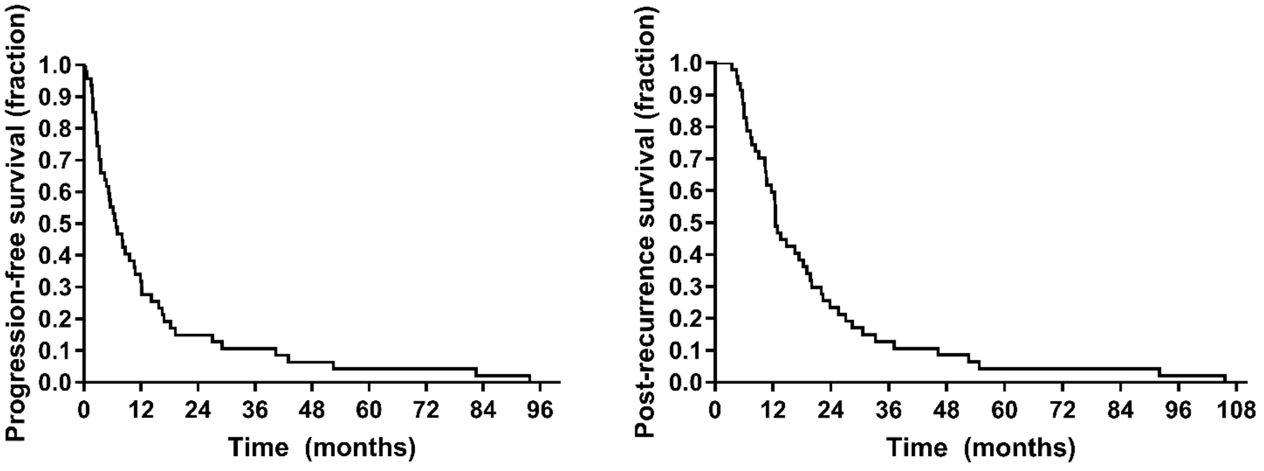

(a) (b)

Supplementary figure S1. Survival curves. (a) Time-to-treatment failure (TTF) of the intention to treat population after iPDT treatment; (b) post-recurrence survival (PRS) of the intention to treat population after iPDT treatment.
